# Image Quality Comparison between Digital Breast Tomosynthesis Images and 2D Mammographic Images Using the CDMAM Test Object

**DOI:** 10.3390/jimaging8080223

**Published:** 2022-08-21

**Authors:** Ioannis A. Tsalafoutas, Angeliki C. Epistatou, Konstantinos K. Delibasis

**Affiliations:** 1 Occupational Health and Safety Department, Radiation Safety Section, Hamad Medical Corporation, Doha P.O. Box 3050, Qatar; 2 Department of Computer Science and Biomedical Informatics, University of Thessaly, 35131 Lamia, Greece

**Keywords:** digital mammography, digital breast tomosynthesis (DBT), image quality, CDMAM

## Abstract

To evaluate the image quality (IQ) of synthesized two-dimensional (s2D) and tomographic layer (TL) mammographic images in comparison to the 2D digital mammographic images produced with a new digital breast tomosynthesis (DBT) system. **Methods:** The CDMAM test object was used for IQ evaluation of actual 2D images, s2D and TL images, acquired using all available acquisition modes. Evaluation was performed automatically using the commercial software that accompanied CDMAM. **Results:** The IQ scores of the TLs with the in-focus CDMAM were comparable, although usually inferior to those of 2D images acquired with the same acquisition mode, and better than the respective s2D images. The IQ results of TLs satisfied the EUREF limits applicable to 2D images, whereas for s2D images this was not the case. The use of high-dose mode (H-mode), instead of normal-dose mode (N-mode), increased the image quality of both TL and s2D images, especially when the standard mode (ST) was used. Although the high-resolution (HR) mode produced TL images of similar or better image quality compared to ST mode, HR s2D images were clearly inferior to ST s2D images. **Conclusions:** s2D images present inferior image quality compared to 2D and TL images. The HR mode produces TL images and s2D images with half the pixel size and requires a 25% increase in average glandular dose (AGD). Despite that, IQ evaluation results with CDMAM are in favor of HR resolution mode only for TL images and mainly for smaller-sized details.

## 1. Introduction

Digital mammography has many advantages over classic screen-film mammography, due to the wide dynamic range and the processing capabilities of digital mammography systems, especially for the dense/glandular breasts of younger women [1]. The advent of Digital Breast Tomosynthesis (DBT) further enhanced the benefits of digital mammography over classic mammography and storage phosphor plate (CR)-based digital mammography [2,3,4,5,6]. DBT systems acquire a number of 2D projections, while the X-ray tube moves in an arc around 0 degrees (perpendicular to the detector, or Z-axis), sweeping an angle ranging from ±12.5 degrees up to ±25 degrees. The sweep angle and the movement/exposure mode vary between manufacturers (continuous or step-and-shoot). The transmission data from the DBT data set are processed to produce 2D tomographic layer (TL) images, parallel to the detector level (usually 1 mm thick), also referred to as DBT slices or focal planes. In this way, any existing lesions in the various layers can be imaged in focus, while the over- and underlying structures are blurred, thus increasing the detection efficiency. Additionally, a synthesized 2D image (also called synthetic 2D; s2D) is generated by post-processing the original DBT data set, which mimics the actual 2D projections acquired in digital mammography [2]. DBT was initially introduced as adjunct to the 2D mammography, but later it was proposed that DBT can replace one of the 2D views (the mediolateral) or even both [2,7,8,9]. The original idea behind s2D images was that achieving similar image quality (IQ) to the actual 2D images would dispense with the need to perform 2D mammography in addition to DBT. It should be mentioned that the dose of DBT is comparable to the dose of 2D mammography; thus, abolishing the need for the latter would be very beneficial for the patient.

A variety of image metrics and phantoms have been employed to quantify the IQ achieved in clinical practice with the various available mammography systems. The phantoms for digital mammography systems introduced by the American College of Radiology (ACR) are still used as an IQ benchmark for assessing 2D screening of a mammography system [10,11]. However, for more elaborate IQ evaluations, the use of the CDMAM phantom is considered as the gold standard [12,13]. According to the study of Mackenzie et al. [14], the clinical effectiveness of mammography for the task of detecting calcification clusters was found to be correlated with the IQ assessment using the CDMAM phantom. Therefore, it was concluded that IQ assessment using CDMAM is justified as a surrogate for assessing the cancer detection performance of mammography systems. However, it should be noted that for non-calcification lesion detection, such a correlation was not established [14].

Regarding IQ evaluation in DBT, the Protocol for the Quality Control of the Physical and Technical Aspects of Digital Breast Tomosynthesis Systems [15] (henceforth referred to as the DBT QC protocol) acknowledges that the current phantoms designed for evaluation IQ in 2D mammography cannot be used to assess image reconstruction. Furthermore, they should not be used for performance comparisons between different models, because they do not include mammographic backgrounds and exhibit disadvantages when used on DBT systems. However, it is also acknowledged that, until 3D phantoms have been developed and validated, the current 2D phantoms can be used for stability assessment and quantification of some aspects of IQ in DBT. In the same document, it is specifically noted regarding CDMAM that: (a) although the IQ evaluation results of CDMAM DBT images have not been extensively validated (since the methods and software used to convert automated analysis into predicted human values are validated for 2D images only), such evaluation of DBT images may be a useful interim tool for monitoring the IQ stability of DBT; (b) CDMAM DBT images may require special processing before automated reading; and (c) the EUREF performance limits for 2D systems are not applicable for DBT [15]. Despite these reservations, a number of published reports (e.g., the NHS Breast Screening Programme Equipment Report series) have used CDMAM for the evaluation of DBT images in the same way as in 2D mammography systems.

In the present study, the CDMAM phantom and its accompanying software were used for IQ evaluation of a new DBT mammography system, in all 2D and DBT acquisition modes available for clinical use. The results of IQ evaluation of 2D projections, and s2D and TL images, were compared to the relevant EUREF acceptable and achievable limit values [12].

## 2. Materials and Methods

### 2.1. CDMAM Phantom Description

**Figure 1:**
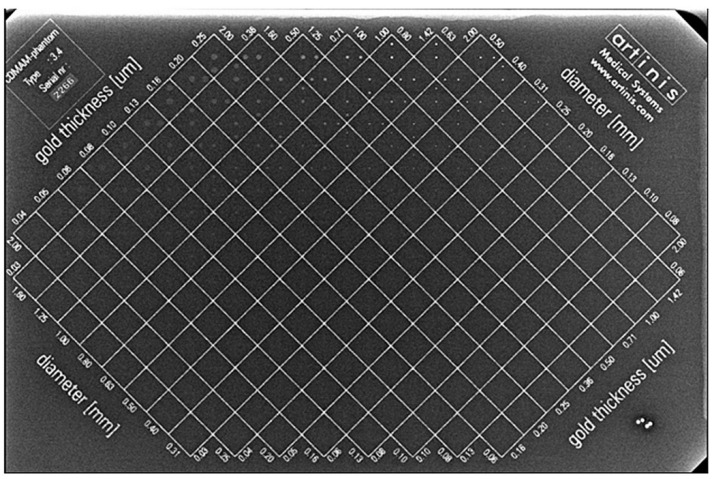
Radiographic appearance of the CDMAM phantom used in this study.

The CDMAM version 3.4 phantom (Artinis Medical Systems, Elst, The Netherlands), whose radiographic appearance is shown in Figure 1, consists of a 0.5 mm thick aluminum base, on which are attached gold disks of various thicknesses (0.03 to 2 μm) and diameters (0.06 to 2 mm), and is enclosed in a PMMA cover. Starting from the upper left corner of the image, a matrix rotated by −45 degrees contains columns with gold disks of constant thickness and progressively smaller diameters, and rows with constant diameters and progressively increasing thickness. Each one of the 205 square matrix elements contains two disks: one in the center of the square and one in the periphery. The peripheral disks are located in one of the 4 corners of each of the matrix elements, following a random pattern. The phantom is enclosed between two 4-Polymethylmethacrylate (PMMA) plates of 1 cm thickness, with dimensions 18 cm × 24 cm. The phantom is considered equivalent to 5 cm of PMMA and 6 cm of compressed breast (50% glandular−50% adipose). More details about the phantom characteristics can be found in the phantom user manual and relevant literature [13,16].

### 2.2. Image Quality Evaluation Using the CDMAM Phantom

**Figure 2:**
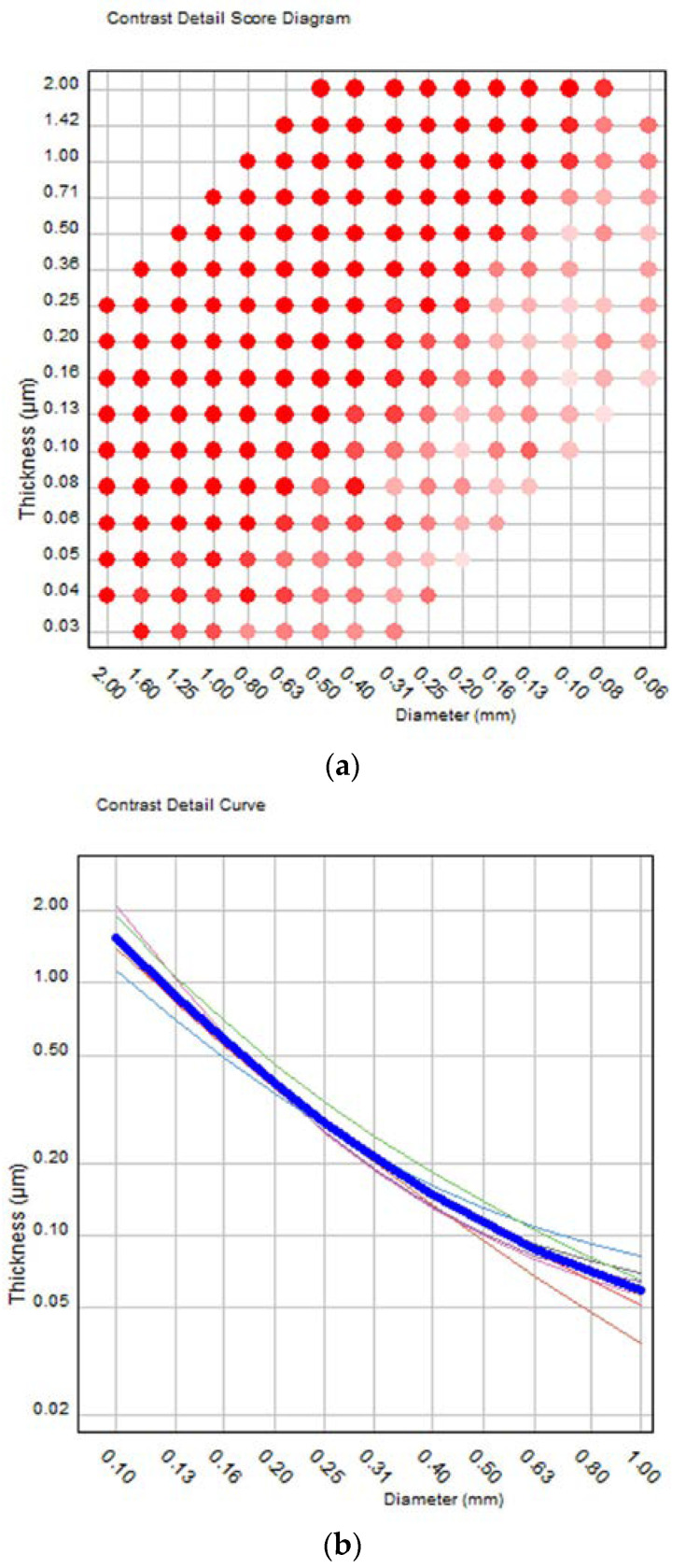

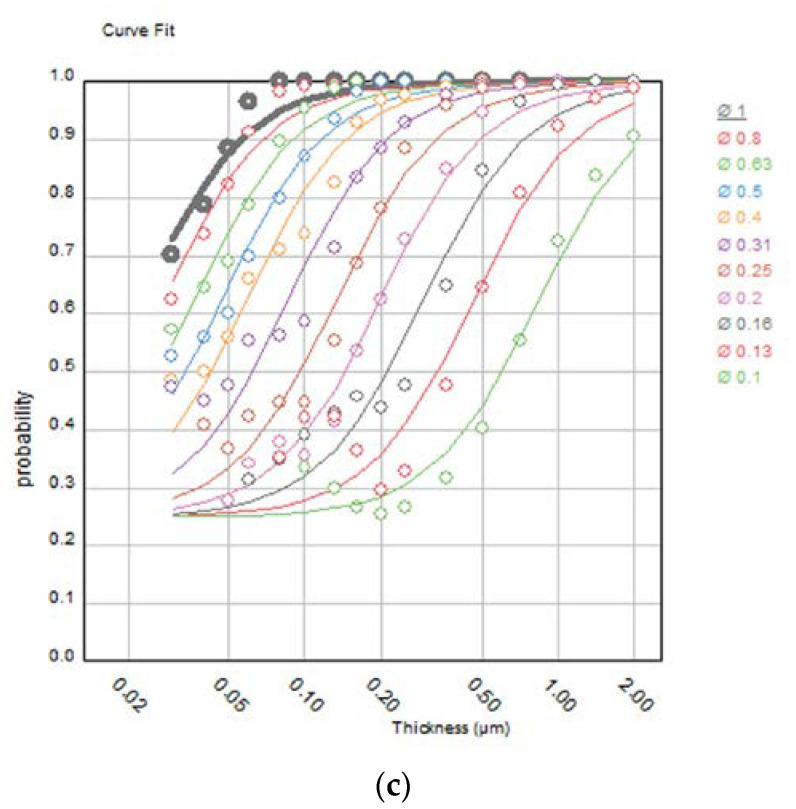
Examples of the output of the CDMAM3.4 Analyser software: (**a**) contrast–detail score diagram, (**b**) contrast–detail curves for each individual image (thin colored lines) and the average curve (thick blue line) for all images, (**c**) average psychometric detection probability (data points and fitted curves) for all images in relation to gold disk thickness and disk diameter.

The main task of the CDMAM phantom is to correctly detect the peripheral disk in each square matrix element by selecting the correct corner of the square (thus, there is a 25% probability that this may be done correctly by chance). For the automatic scoring of digital phantom images, the phantom manufacturer offers the CDMAM 3.4 Analyser software v2.3 (henceforth called “software”), with a very user-friendly graphical user interface (GUI). After importing the images in DICOM format and adjusting the image rotation and pixel intensity relationship sign (if default values do not work), automated scoring of all images together or one by one is performed. It must be mentioned that image quality scores with the CDMAM phantom may vary depending on the relative position of the phantom’s gold disks in respect to the image receptor elements. For this reason, 8 to 16 images should be acquired for each acquisition protocol and CDMAM should be slightly moved between exposures. The automatic scoring of the phantom produces 4 basic outputs, an example of which is shown in Table 1 and Table 2 and Figure 2a–c.

The first output consists of two tables. The first table (see Table 1) contains the values of the image quality figure *IQF_inv_* and total detected scores for each individual image and respective average scores (for all images). The *IQF_inv_* is defined by the following equation:

(1) $$IQF_{inv}=\frac{100}{\sum_{i=1}^{16}t_{thr,i}\times d_{i}}$$

where, for each column *i* (of the 16 columns) of the phantom, with diameter *d_i_, t_thr,i_* is the respective threshold gold thickness. For completely visible or invisible columns, the smallest or the largest disk diameter is used, respectively. Smaller threshold thickness values, which denote better IQ, decrease the denominator value, thus increasing the value of *IQF_inv_*.

The second table presents the average threshold values of gold thickness (automatic, predicted human, and fit-to-predicted human) in relation to the gold disk diameters (Figure 2a). For IQ evaluation the fit-to-predicted human threshold (last row of the table) is used, especially the values for 0.1, 0.25, 0.5, and 1 mm diameter disks. These values are compared with the acceptable and achievable values given by EUREF as performance limits, which have been set for the above four disk diameters, as presented in Table 3. The smaller the threshold gold thickness, the better the IQ [14].

The second output (shown in Figure 2a) is the contrast detail score diagram which consists of a gridline representing the matrix of the phantom, with red dots, pink dots, and vacant gridline intersection positions, which denote respectively the correct detection of both the central and peripheral disk, only one of them, and neither of them. The number of red dots expressed as a percentage of the total number of squares (205 plus the 2 missing corners of the phantom, 0.03 μm/2 mm and 2 μm/0.06 mm, which are counted as detected when both their neighbors are detected) is the total detected score (%) shown in Table 1.

The third output (shown in Figure 2b) is a graph with the contrast–detail curves for each individual image (thin colored lines) and the respective average curve (thick blue line) for all images. The fourth (shown in Figure 2c) is the average psychometric detection probability (data points and fitted curves) for all images in relation to gold disk thickness, for disk diameters from 0.1 to 1 mm. More details about the software, the scoring procedure, and the theoretical background of the IQ evaluation with CDMAM can be found in the referenced literature [12,13,16,17,18,19,20].

### 2.3. Mammography System and Acquisition Modes

The mammography system evaluated was a Fujifilm Amulet Innovality (Software version: FDR-3000 AWS V9.1). This specific model was recently installed in a public hospital in Greece and, unlike its predecessor model, it allows DBT acquisitions with both high-resolution (HR) mode and standard (ST) resolution mode, using iterative reconstruction algorithms (ISR) for s2D images and TL image formation. In ST mode, the sweep angle is 15 degrees (−7.5° to 7.5°) and the pixel size of both s2D and TL images is 100 μm, whereas in HR mode, the sweep angle is 40 degrees (−20° to 20°) and the pixel size of both s2D and TL images is 50 μm (the same as in 2D acquisition mode). For both ST and HR DBT acquisition modes and for the 2D acquisition mode, two dose modes are available: the N-mode (normal dose) and the H-mode (high dose).

Sets of eight 2D images were acquired using the N-mode, H-mode, and the four DBT modes (N-mode (ST), H-mode (ST), N-mode (HR), and H-mode (HR)) available for the clinical practice. All images were acquired with the small compression paddle (18 cm × 24 cm). It must be noted that TL and s2D images of the CDMAM phantom from the DBT system were scored in the original format and no additional processing was applied, so as to reflect the IQ using the same processing conditions as those in clinical practice. For all images, the mammography system information, technical parameters, and exposure conditions reported later in the figure legends and the table were derived using free software named DICOM Info Extractor, which facilitates the automatic extraction of the DICOM header information [21].

To investigate the impact of the compression paddle height setting on exposure factors and breast average glandular dose (AGD) in Fujifilm Amulet Innovality and the effect of field size, two additional sets of CDMAM images were acquired using the auto 2D and DBT acquisition modes (only 1 exposure per acquisition mode) with the compression paddle positioned at 60 and 45 mm, and one more set (only 1 exposure per acquisition mode) with the large compression paddle (24 cm × 30 cm) positioned at 60 mm.

## 3. Results

**Figure 3:**
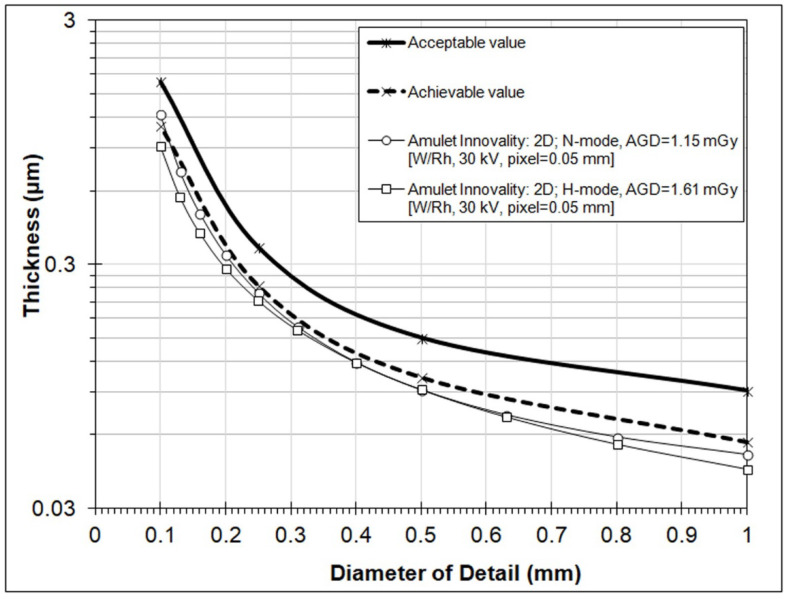
Contrast–detail curves for 2D images obtained using two dose levels (N-mode and H-mode). (Results from the CDMAM3.4 Analyser software).

**Figure 4:**
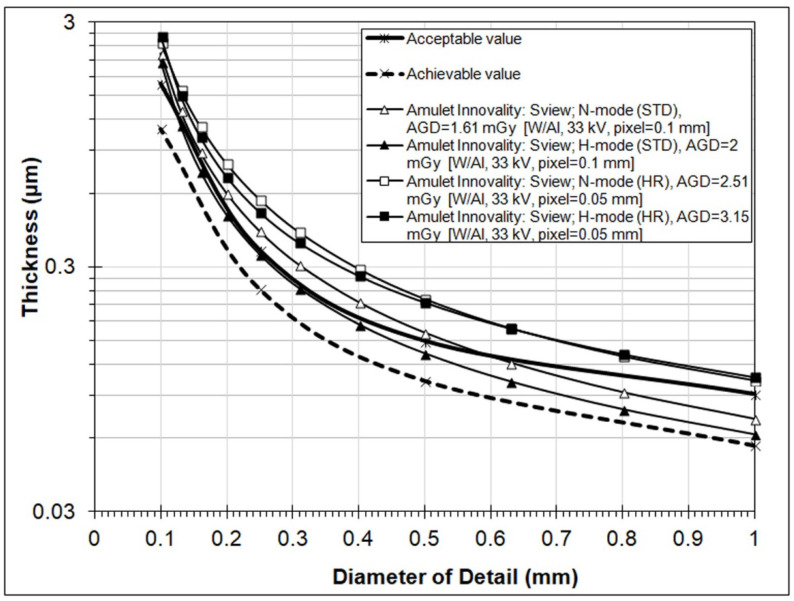
Contrast–detail curves for s2D (S-view) images obtained using four different operation modes (N-mode (ST), H-mode (ST), N-mode (HR,) and H-mode (HR)) for DBT acquisitions. (Results from the CDMAM3.4 Analyser software).

**Figure 5:**
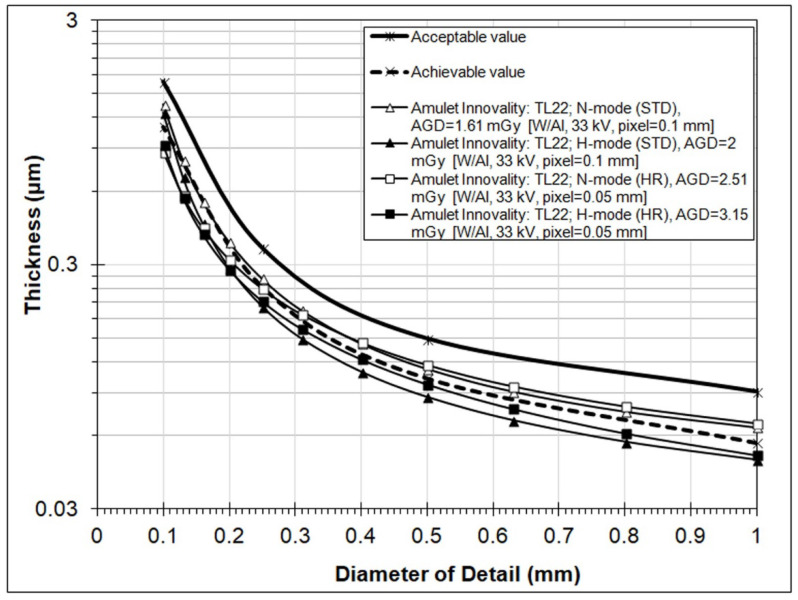
Contrast–detail curves for tomographic images (TL22) obtained using four different operation modes (N-mode (ST), H-mode (ST), N-mode (HR), and H-mode (HR)) for DBT acquisitions. The curve for H-mode (HR) is based on a single image. (Results from the CDMAM3.4 Analyser software).

In the following, the results of IQ evaluation of the new Fujifilm Amulet Innovality DBT system are reported in terms of the fit-to-predicted human gold thickness (TFit) values. These are shown in Figure 3, Figure 4 and Figure 5 and in Table 4, where, along with IQ evaluation results, the exposure factors, AGD, and pixel size of the 2D and DBT images are reported. All acquisitions were performed with the compression paddle set at 60 mm and manually selected exposure factors to match the respective exposure factors selected by the AEC system, for imaging 50 mm of PMMA plates with the compression paddle set at 60 mm.

Figure 3 shows the contrast–detail curves obtained from the 2D images. It can be observed that the curves obtained using H-mode and N-mode practically coincide for detailed diameters in the range 0.3 to 0.6 mm. However, it is obvious that H-mode offers better IQ according to the respective *IQF_Inv_* and total detected values, as shown in Table 4. Both curves lie below the achievable EUREF curves (with the exception of the first point of the curve for N-mode).

Figure 4 depicts the contrast–detail curves obtained from the s2D (called S-view) images. It is apparent that s2D images have inferior IQ, as only H-mode (ST) nearly satisfied the EUREF acceptable value limits (except for the first data point, namely for 0.1 mm diameter details). It is noticeable that HR mode produced s2D images of lower quality (larger threshold thicknesses) than ST mode, for both N-mode and H-mode. Again, an increase in IQ scores using H-mode was observed, compared to N-mode, which was true irrespectively of the disk diameter size, only for ST resolution mode. The respective *IQF_Inv_* and total detected values shown in Table 4 verified that HR s2D images are inferior to ST s2D images and that the increase in IQ using H-mode is more pronounced for ST mode.

Finally, Figure 5 shows the contrast–detail curves obtained from the TL (tomographic layer) images. It must be noted that for each DBT acquisition mode, at least five TL images around the actual position of the CDMAM phantom aluminum base were scored. The results made evident that the phantom’s base was best focused at a height of 22 mm above the breast support table (TL22), which corresponded to the 23rd image of the DBT image set, since image numbering starts from the TL image that corresponds to a layer height of 0 mm (the surface of the support table). Scores were maximum for the TL22 images and deteriorated for layer images above or below this plane.

In Figure 5, it can be seen that TL images had very good IQ, as all four curves satisfied the EUREF acceptable value limits. In fact, a few scores, e.g., for H-mode (ST) and for diameters 0.25 and 0.5 mm, were even better than the respective scores of the 2D images. Furthermore, and partially in contrast to what was observed for s2D images, for TL images the HR resulted in reduced threshold thicknesses (i.e., increased detectability) compared to the ST mode, by ~30% for disk diameter 0.1 mm (both for N- and H-modes) and 10% for disk diameter 0.25 mm (N-mode). For other gold disk diameters, the HR mode resulted in increased threshold disk thicknesses, by up to about 17%. For TL images obtained with standard resolution, the H-mode resulted in bigger IQ scores compared to the N-mode. A similar trend was observed for HR mode, except for the smallest disk diameters, where the H-mode resulted in a slightly larger threshold thickness than that with the N-mode. The respective *IQF_Inv_* and total detected values shown in Table 4 verified that IQ of TL increases with H-mode for both resolution modes, and suggested that TL images with HR are superior to those obtained with ST mode.

**Figure 6:**
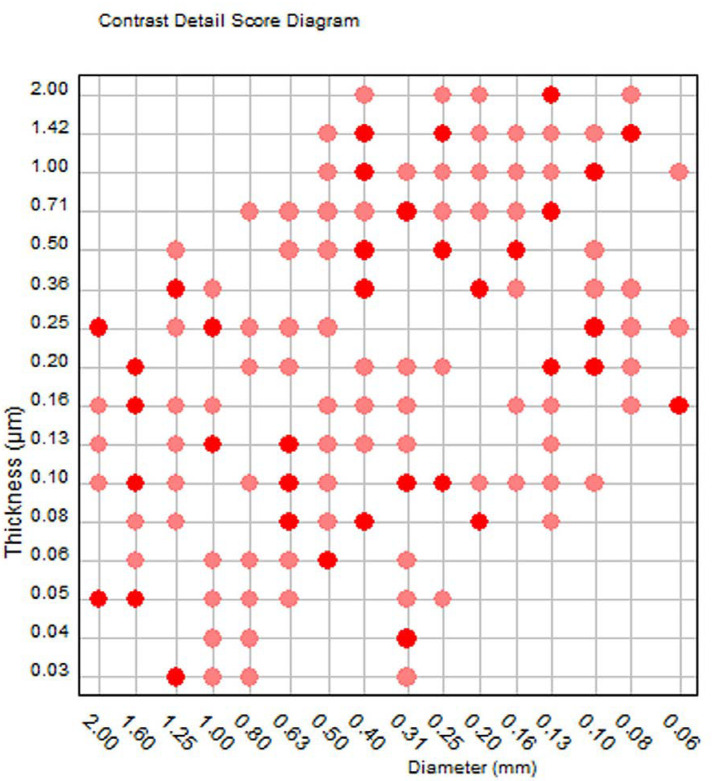
An example of erratic contrast–detail score diagram output obtained for TL22 images obtained with H-mode (HR) operation mode (output of the CDMAM3.4 Analyser software). It can be seen that larger and thicker disks are not detected but smaller and thinner disks are.

As mentioned in the footnote of Table 4 and the legend of Figure 5, the results for H-mode (HR) are based on only one image. The remaining seven images of the set produced erratic results, an example of which is shown in Figure 6. Unlike the contrast detail score diagram shown in Figure 2b, where, as expected, both the central and peripheral disks of larger thicknesses and diameters are detected first, and disks of smaller diameters and thicknesses are progressively missed, in Figure 6 disk detection follows a rather random pattern. The reason why these images were rejected could not be explained. It was initially thought that this could be attributed to wrong phantom positioning in the small field, but visually the images were perfect, gold details were conspicuous, and they were no missing areas of the phantom. Moreover, it was rather strange that the respective images at the other focal planes (i.e., TL20, TL21, TL23, and TL24) did not produce erratic results. However, since the same problem was observed with the TL22 image acquired with H-mode (HR) and the 24 cm × 30 cm compression paddle, it became clear that the problem was not the field size. It must be noted that the results of the single H-mode (HR) image scores were considered reliable, because similar results were obtained for two more images acquired with auto-dose mode and the compression paddle positioned at 45 and 60 mm.

From the additional sets of CDMAM images acquired using the auto modes, it was seen that, for CBT = 60 mm, the automatically selected exposure factors with CDMAM were practically identical (the mAs were only 2–4% less) as those determined using 50 mm PMMA and CBT = 60 mm. Therefore, in this DBT system, the CDMAM does not increase the exposure factors and CDMAM phantom images can be also acquired using the AEC mode. With CBT set at 45 mm, the automatically selected kVp was 1 kV less than that with CBT = 60 mm, for both 2D and DBT acquisitions. For 2D acquisitions, the mAs and AGD values were respectively 27% and 31% larger. By comparison, for the DBT acquisition modes, the mAs and AGD values were only increased by 1 to 5%.

## 4. Discussion

Concerning the DBT images, it was seen that the IQ of tomographic layers of the CDMAM phantom is, in general, comparable (although usually slightly inferior) to that of 2D images and satisfies the EUREF acceptable value limits. However, the IQ of s2D (S-view) images was lower than that of TL images and did not satisfy the EUREF limits. A noticeable observation was that although HR acquisition mode, in comparison with ST mode, resulted in images with half the pixel size, it worsened the IQ of s2D images; in contrast, for TL images, improvement was seen only in the detection of smaller disk diameters (<0.4 mm for N-mode and <0.2 mm for H-mode). It must be also noted that for DBT acquisitions with H-mode (HR), the AGD is 3.15 mGy (as can be seen in Table 4), higher than the limiting value of 3 mGy for DBT (60 mm breast) [15]. Finally, it was seen that the use of H-mode (ST) instead of N-mode (ST) results in better quality 2D images (for disk diameters <0.5 mm), and better s2D and TL images (for all disk diameters), at the expense of an increase in AGD (40% for 2D and 24% for DBT).

Table 4 shows that the two additional IQ indices calculated by the software, *IQF_Inv_* and total detected (%), are both larger in H-mode than in N-mode, and increase in HR mode compared to ST mode for TL images but decrease for s2D images. Overall, larger *IQF_Inv_* and total detected values were observed for the 2D images produced with H-mode and the second-largest values for TL images acquired with H-mode (HR).

As previously mentioned, in the DBT QC protocol, concerns about the suitability of the CDMAM phantom for IQ evaluation of DBT images have been expressed [15]. Therefore, the results of this study should be interpreted with caution and are not indented to be used to demote the actual diagnostic IQ of TL or s2D images. However, the reason that s2D images and, in part, tomographic images are inferior to the original 2D images of the CDMAM could be attributed to the fact that s2D images and tomographic images are the result of complex reconstruction procedures of the DBT data set, which inevitably introduce some inaccuracies, unlike the 2D projections, the production of which is quite straightforward.

Concerning the performance comparison of s2D and 2D images, in a study by Stacampiano et al. [22], where the CDMAM phantom was used, it was shown that the IQ of s2D images from a Hologic DBT system (called c-view) were clearly inferior to the IQ of 2D images. Indeed, the contrast–detail curve for s2D images was well above the acceptable EUREF curve, whereas for 2D images, most parts of the contrast–detail curve were below the achievable EUREF curve. In the same study, the IQ inferiority of s2D compared to 2D images was also documented using other phantoms. Nelson et al. [23], using the ACR and a novel 3D anthropomorphic phantom, concluded that s2D images from a Hologic Selenia Dimensions DBT system, although providing enhanced visualization of medium and large microcalcification objects, provided poorer overall resolution and noise properties. Indeed, it was reported that 50% to 70% of ACR phantom images failed to satisfy the ACR accreditation requirements, primarily due to fiber breaks. The results of both of these studies are in agreement with the results of the present study. In contrast, Wahab et al. [24], based on the results of a comparison of FFDM (2D) and s2D images of actual breast images, concluded that radiologists interpreting s2D and FFDM digital mammography images have similar frequencies of detection of calcifications and BIRADS assessment, and, therefore, a synthetic 2D mammogram may be a sufficient replacement for FFDM at screening.

Digital 2D mammography is the current standard, as far as screening mammography is concerned, but DBT is gaining ground in clinical practice and there have been many studies presenting the benefits of DBT in the detection of cancer over 2D mammography, based on some of which, FDA approval was initially granted for the use of DBT in clinical practice [2]. However, the evolution of DBT continues and some manufacturers have already incorporated iterative reconstruction techniques (as in the DBT system evaluated in this study) instead of filtered back projection, to improve the IQ of tomographic and s2D images [2]. Since most radiologists have been trained in and are accustomed to relying on 2D images for diagnosis, the need to meet the demand for high-quality s2D images remains imperative. However, it should be stressed that s2D images are not intended to be a standalone examination like 2D mammography and should be always interpreted along with the tomographic layer images [2].

Although, in this study, s2D images (and partly TL images) of the CDMAM phantom exhibited inferior IQ compared to the respective 2D images, this does not mean that DBT alone may not be adequate for diagnosis. Unlike the CDMAM phantom, where all the details are found within a layer of just 0.5 mm, real breasts contain structures critical for diagnosis that extend over several layers within the compressed breast. Therefore, the diagnostic benefits that arise from the separation of superimposing layers in clinical practice, in comparison to 2D mammography, cannot be fully assessed with the CDMAM phantom. However, the fact that, in this study, TL images exhibited better IQ scores than the s2D images (although both were produced utilizing the same DBT data set), is an indication of such an advantage of tomographic images.

Finally, it is worth mentioning that image enhancement techniques based on deep learning have started to emerge. For instance, a very recent report [25] describes the utilization of a convolutional neural network (CNN) for image denoising, based on PCA sparsity estimation, which has been applied to cerebral microbleed detection in susceptibility weighted magnetic resonance images. Despite the effectiveness of similar methods on certain imaging modalities, the purpose of our work was to assess the quality of phantom mammographic images acquired under conditions identical to the acquisition of clinical images. The possible application of several image enhancement algorithms on clinical s2D and DBT images of all kinds of lesions (cancerous, non-calcified, etc.) and the subsequent measurable effect on the quality of the CDMAM phantom images is very important and requires extensive further work.

## 5. Conclusions

The automatic evaluation of CDMAM phantom images acquired with a DBT system demonstrated that 2D images exhibit better IQ than synthesized 2D images and, in most cases, than tomographic images. Tomographic layers clearly exhibited better IQ than synthesized 2D images and satisfied the EUREF limits, unlike the synthesized 2D images, which presented inferior IQ compared to the EUREF requirements; these requirements are currently applicable only for actual 2D projections. For both TL and s2D images, improvement in IQ was observed when H-mode was used instead of N-mode. In contrast to expectations, HR mode only resulted in improvement in IQ in TL images, and mainly for small diameter-sized details, whereas for large-diameter details, the opposite effect was observed. Furthermore, HR mode produced inferior s2D images compared to ST mode for all detail sizes.

## Figures and Tables

**Table 1:** Example of the output of the CDMAM3.4 Analyser software: *IQFinv* and Total detected scores for each image and the average of whole images (Group score).

Image Number	*IQF_inv_*	Total Detected (%)
1	148.19	67.61
2	118.69	65.34
3	144.99	67.61
4	143.48	68.18
5	144.94	69.32
6	159.34	69.89
7	135.80	68.47
8	141.95	67.61
**Group Score**	**141.59**	**68.00**

**Table 2:** Example of the output of the CDMAM3.4 Analyser software: average scores (from all images) in terms of threshold values of thickness (automatic, predicted human and fit-to-predicted Human) for the gold disk diameters.

	Gold Disk Diameters (mm)										
Score	0.10	0.13	0.16	0.20	0.25	0.31	0.40	0.50	0.63	0.80	1
*T_auto_* ^1^	0.839	0.463	0.292	0.197	1.133	0.086	0.059	0.047	0.037	0.028	0.023
*T_pred_* ^2^	1.231	0.723	0.479	0.337	0.237	0.160	0.114	0.094	0.075	0.585	0.049
*T_Fit_* ^3^	1.240	0.723	0.488	0.329	0.229	0.167	0.119	0.092	0.072	0.590	0.050

^1^ Automatic threshold gold thickness (μm). ^2^ Predicted human gold thickness (μm). ^3^ Fit-to-predicted human gold thickness (μm).

**Table 3:** Acceptable and achievable EUREF performance limits.

Gold Disk Diameter (mm)	Acceptable (μm)	Achievable (μm)
1	0.091	0.056
0.5	0.15	0.103
0.25	0.352	0.244
0.1	1.68	1.1

**Table 4:** The results of automatic image quality evaluation of CDMAM images acquired with the Amulet Innovality DBT mammography unit, along with information about the exposure parameters and average glandular dose (AGD) values, as calculated by the mammography unit. Bold italics denote values above the EUREF acceptable values.

Data Source	Pixel (mm)	AGD (mGy)	Anode/Filter, kVp, mAs	Fit to Predicted Human Threshold Gold Thickness (in μm) for Detail Diameters (mm)				*IQF_Inv_*	Total Detected (%)
				0.1	0.25	0.5	1		
2D: N-mode	0.05	1.15	W/Rh, 30, 71	1.23	0.23	0.09	0.05	141.59	68
2D: H-mode	0.05	1.61	W/Rh, 30, 100	0.91	0.21	0.09	0.04	162.24	69.7
Sview: N-mode (ST)	0.1	1.61	W/Al, 33, 32	***2.24***	***0.42***	***0.16***	0.07	80.26	58.84
Sview: H-mode (ST)	0.1	2	W/Al, 33, 40	***2.08***	0.34	0.13	0.06	94.28	61.65
Sview: N-mode (HR)	0.05	2.51	W/Al, 33, 50	***2.49***	***0.57***	***0.22***	***0.1***	62.16	53.44
Sview: H-mode (HR)	0.05	3.15	W/Al, 33, 63	***2.63***	***0.5***	***0.22***	***0.11***	64.92	54.08
TL22: N-mode (ST)	0.1	1.61	W/Al, 33, 32	1.36	0.26	0.11	0.06	121.43	65.48
TL22: H-mode (ST)	0.1	2	W/Al, 33, 40	1.26	0.2	0.09	0.05	150.45	68.61
TL22: N-mode (HR)	0.05	2.51	W/Al, 33, 50	0.87	0.24	0.12	0.07	139.56	67.22
TL22: H-mode (HR) *	0.05	3.15	W/Al, 33, 63	0.93	0.21	0.1	0.05	157.32	69.32

* Results are based in a single image.

## Data Availability

Not applicable.

## References

[1] Pisano E.D., Gatsonis C., Hendrick E., Yaffe M., Baum J.K., Acharyya S., Conant E.F., Fajardo L.L., Bassett L., D’Orsi C. (2005). Diagnostic performance of digital versus film mammography for breast-cancer screening. N. Engl. J. Med..

[2] Durand M.A. (2018). Synthesized Mammography: Clinical Evidence, Appearance, and Implementation. Diagnostics.

[3] Hovda T., Holen Å.S., Lång K., Albertsen J.L., Bjørndal H., Brandal S.H., Sahlberg K.K., Skaane P., Suhrke P., Hofvind S. (2020). Interval and Consecutive Round Breast Cancer after Digital Breast Tomosynthesis and Synthetic 2D Mammography versus Standard 2D Digital Mammography in BreastScreen Norway. Radiology.

[4] Hofvind S., Holen Å.S., Aase H.S., Houssami N., Sebuødegård S., Moger T.A., Haldorsen I.S., Akslen L.A. (2019). Two-view digital breast tomosynthesis versus digital mammography in a population-based breast cancer screening programme (To-Be): A randomised, controlled trial. Lancet Oncol..

[5] Bahl M., Mercaldo S., Vijapura C.A., McCarthy M.A., Lehman C.D. (2019). Comparison of performance metrics with digital 2D versus tomosynthesis mammography in the diagnostic setting. Eur. Radiol..

[6] Alshafeiy T.I., Nguyen J.V., Rochman C.M., Nicholson B.T., Patrie J.T., Harvey J.A. (2018). Outcome of Architectural Distortion Detected Only at Breast Tomosynthesis versus 2D Mammography. Radiology.

[7] Romero Martín S., Raya Povedano J.L., Cara García M., Santos Romero A.L., Pedrosa Garriguet M., Álvarez Benito M. (2018). Prospective study aiming to compare 2D mammography and tomosynthesis + synthesized mammography in terms of cancer detection and recall. From double reading of 2D mammography to single reading of tomosynthesis. Eur. Radiol..

[8] Tagliafico A.S., Calabrese M., Bignotti B., Signori A., Fisci E., Rossi F., Valdora F., Houssami N. (2017). Accuracy and reading time for six strategies using digital breast tomosynthesis in women with mammographically negative dense breasts. Eur. Radiol..

[9] Kang H.-J., Chang J.M., Lee J., Song S.E., Shin S.U., Kim W.H., Bae M.S., Moon W.K. (2016). Replacing single-view mediolateral oblique (MLO) digital mammography (DM) with synthesized mammography (SM) with digital breast tomosynthesis (DBT) images: Comparison of the diagnostic performance and radiation dose with two-view DM with or without MLO-DBT. Eur. J. Radiol..

[10] ACR (1999). Mammography Quality Control Manual.

[11] ACR (2018). Digital Mammography Quality Control Manual.

[12] EUREF (2006). European Protocol for the Quality Control of the physical and technical aspects of mammography screening. European Guidelines for Quality Assurance in Mammography Screening.

[13] EUREF (2013). European Guidelines for Quality Assurance in Mammography Screening.

[14] Mackenzie A., Warren L.M., Wallis M.G., Given-Wilson R.M., Cooke J., Dance D.R., Chakraborty D.P., Halling-Brown M.D., Looney P.T., Young K.C. (2016). The relationship between cancer detection in mammography and image quality measurements. Phys. Med..

[15] van Engen R.E., Bosmans H., Bouwman R.W., Dance D.R., Heid P., Lazzari B., Marshall N., Phelan N., Schopphoven S., Strudley C. (2018). Protocol for the Quality Control of the Physical and Technical Aspects of Digital Breast Tomosynthesis Systems, Version 1.03.

[16] Artinis (2017). Manual Contrast-Detail Phantom CDMAM 3.4 & CDMAM 3.4 Analyser Software V2.3, Version 1.3.

[17] Young K., Alsagera A., Oduko J.M., Bosmans H., Verbrugge B., Geertse T., van Engen R. (2008). Evaluation of software for reading images of the CDMAM test object to assess digital mammography systems. Proceedings Volume 6913, Medical Imaging 2008: Physics of Medical Imaging 69131C.

[18] Veldkamp W.J., Thijssen M.A., Karssemeijer N. (2003). The value of scatter removal by a grid in full field digital mammography. Med. Phys..

[19] Figl M., Hoffmann R., Kaar M., Semturs F., Brasik N., Birkfellner W., Homolka P., Hummel J. (2011). Factors for conversion between human and automatic read-outs of CDMAM images. Med. Phys..

[20] Figl M., Homolka P., Osanna-Elliott A., Semturs F., Kaar M., Hummel J. (2016). Conversion factors between human and automatic readouts of CDMAM phantom images of CR mammography systems. Phys. Med. Biol..

[21] Tsalafoutas I.A., Metallidis S. (2011). A method for calculating the dose length product from CT DICOM images. Br. J. Radiol..

[22] Stancampiano C., Boschiroli L., Campoleonic M., Vismarab L.O. (2018). Comparison of 2D synthesized mammography versus standard full-field digital mammography: Quantitative and qualitative image analysis. Phys. Med..

[23] Nelson J.S., Wells J.R., Baker J.A., Samei E. (2016). How does c-view image quality compare with conventional 2D FFDM?. Med. Phys..

[24] Wahab R.A., Lee S.J., Zhang B., Sobel L., Mahoney M.C. (2018). A comparison of full-field digital mammograms versus 2D synthesized mammograms for detection of microcalcifications on screening. Eur. J. Radiol..

[25] Liu H., Rashid T., Ware J., Jensen P., Austin T., Nasrallah I., Bryan R., Heckbert S., Habes M. (2021). Adaptive Squeeze-and-Shrink Image Denoising for Improving Deep Detection of Cerebral Microbleeds. International Conference on Medical Image Computing and Computer-Assisted Intervention.

